# Differential regulation of mTOR signaling determines sensitivity to AKT inhibition in diffuse large B cell lymphoma

**DOI:** 10.18632/oncotarget.7036

**Published:** 2016-01-27

**Authors:** Scott A. Ezell, Suping Wang, Teeru Bihani, Zhongwu Lai, Shaun E. Grosskurth, Suprawee Tepsuporn, Barry R. Davies, Dennis Huszar, Kate F. Byth

**Affiliations:** ^1^ AstraZeneca Oncology, Waltham, Massachusetts, MA, USA; ^2^ AstraZeneca Oncology, Macclesfield, Cheshire, UK

**Keywords:** mTOR, DLBCL, AKT, Ibrutinib, S6K1

## Abstract

Agents that target components of the PI3K/AKT/mTOR pathway are under investigation for the treatment of diffuse large B cell lymphoma (DLBCL). Given the highly heterogeneous nature of DLBCL, it is not clear whether all subtypes of DLBCL will be susceptible to PI3K pathway inhibition, or which kinase within this pathway is the most favorable target. Pharmacological profiling of a panel of DLBCL cell lines revealed a subset of DLBCL that was resistant to AKT inhibition. Strikingly, sensitivity to AKT inhibitors correlated with the ability of these inhibitors to block phosphorylation of S6K1 and ribosomal protein S6. Cell lines resistant to AKT inhibition activated S6K1 independent of AKT either through upregulation of PIM2 or through activation by B cell receptor (BCR) signaling components. Finally, combined inhibition of AKT and BTK, PIM2, or S6K1 proved to be an effective strategy to overcome resistance to AKT inhibition in DLBCL.

## INTRODUCTION

Diffuse large B cell lymphoma (DLBCL) is the most common form of non-Hodgkin lymphoma and is generally classified as either activated B cell (ABC) type or germinal center B cell (GCB) type [1]. ABC-DLBCL is characterized by constitutive BCR and NF-κB signalling, which is required for survival of these tumors [2]. Frequent mutations in the BCR subunits CD79A/B [3] or in NF-κB pathway components CARD11 [4], A20 [5], and MyD88 [6] are responsible for activation of these pathways. GCB tumors often have mutations activating PI3K/AKT/mTOR signalling as well as amplification of the mIR-17-92 locus [7, 8].

Currently, few targeted therapies exist for DLBCL and the standard of care remains chemotherapy combined with the anti-CD20 antibody rituximab (R-CHOP). Preclinical evidence supporting the PI3K/AKT/mTOR pathway as a therapeutic target in DLBCL has recently been presented in a number of studies. The combination of ibrutinib, a specific inhibitor of BTK, with mTOR inhibitors is effective in ABC-DLBCL [9, 10]. The mTORC1 inhibitor everolimus has also been combined with rituximab [11]. In addition, the dual mTOR/PI3K inhibitor NVP-BEZ235 was shown to be effective in GCB-DLBCL both as monotherapy [12] and in combination with the HDAC inhibitor panobinostat [13]. Inhibition of PI3K/AKT signaling through restoration of PTEN expression was also effective in GCB-DLBCL [14]. Most of these studies suggest that differences in sensitivity to pathway inhibition exist between DLBCL subtypes, with most inhibitors of the PI3K/AKT/mTOR pathway showing activity in a subset of GCB-DLBCL. However, a detailed understanding of which subtypes of DLBCL are resistant to inhibitors of component kinases in this cascade is lacking. Furthermore, the mechanism by which resistant cell lines bypass the requirement for AKT has remained elusive.

Here we show that ABC-DLBCL is resistant to treatment with AKT inhibitors (AKTi) despite being sensitive to treatment with mTOR inhibitors. We find that sensitivity to AKTi is regulated at the level of S6K1phosphorylation. In cell lines that are sensitive to AKTi, treatment results in dephopshorylation of S6K1 and S6, whereas resistant cell lines show AKT-independent activation of S6K1/S6 signaling. Hence, S6K1 phosphorylation status in AKTi-treated cells is predictive of response. At least two distinct mechanisms exist to promote AKT-independent activation of S6K1. A subset of ABC-DLBCL shows upregulation of PIM2 which activates S6K1/S6 signaling. Another subset directly activates S6K1 downstream of BCR signaling through a mechanism that bypasses AKT. Ultimately, we use this understanding of PI3K/AKT/mTOR signaling to demonstrate that combined inhibition of AKT and S6K1, PIM2, or BTK can overcome resistance to AKTi alone.

## RESULTS

### Differential sensitivity of DLBCL subtypes to AKT inhibitors

**Figure 1:**
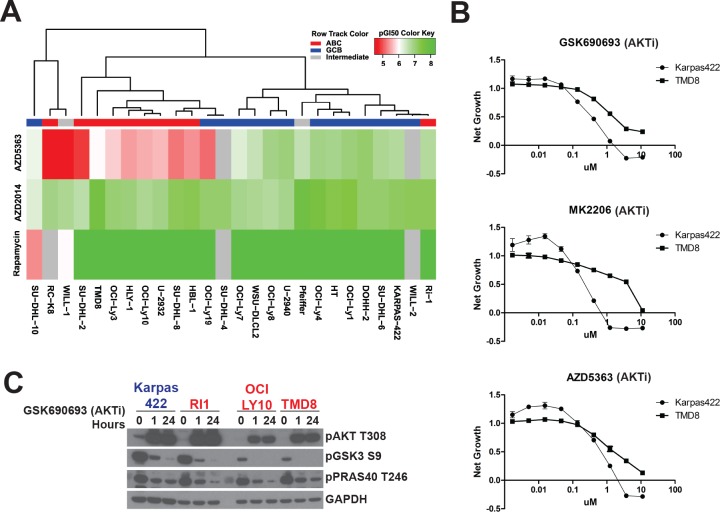
DLBCL subtypes have different sensitivities to AKT inhibitors **A.** Cell lines were sorted according to drug sensitivity (pGI_50_) by unsupervised hierarchical clustering. Sensitivity was determined using a 72h Alamar Blue assay. **B.** Dose response curves were generated for the indicated compounds using a 72h CellTiterGlo assay (*n* = 3). **C.** DLBCL lines were treated with GSK690693 (5μM) for 1h and 24h. ABC cells are colored in red. GCB are colored in blue.

To understand the efficacy of AKT and mTOR inhibitors across DLBCL subtypes, we assembled a panel of DLBCL lines. We assigned each line to the ABC or GCB subtype, or to intermediate status, using gene expression profiling as previously described [15]. We generated dose-response curves for three inhibitors, AZD5363, a catalytic inhibitor of AKT [16], AZD2014, a dual TORC1/2 inhibitor [17], and rapamycin, an allosteric inhibitor of mTORC1, in these cell lines. We calculated pGI_50_ values across the cell lines for each inhibitor and performed an unsupervised hierarchical clustering analysis of the data (Figure 1A). Both mTOR inhibitors showed similar GI_50_ values across all DLBCL lines tested, regardless of subtype. By contrast, AZD5363 showed a wide range of GI_50_ values and primarily drove the clustering. Resistance to AKTi, as defined by a high GI_50_ value (or low pGI_50_), correlated with the ABC subtype. Using a Wilcoxon rank sum test, we compared GI_50_ values between the ABC and GCB subtypes (Supplemental Table 1). Resistance to AZD5363 was strongly correlated with ABC identity (*p* = 0.002) whereas there was no association with sensitivity to rapamycin (*p* = 1.000) or AZD2014 (*p* = 0.963).

We confirmed differential sensitivity to AKTi by selecting for further analysis an AKT-sensitive GCB line, Karpas422, which possesses an inactivating *PTEN* mutation, together with an AKTi-resistant ABC line, TMD8, that carries an activating *CD79B* mutation resulting in constitutive NF-κB activity. We generated dose-response curves for both cell lines with three different AKT inhibitors, AZD5363, GSK690693, and MK2206, the dual TORC1/2 inhibitor AZD2014 and the mTORC1 inhibitor everolimus, using an additional proliferation assay (CellTiterGlo). All three AKT inhibitors showed more potent inhibition of cell proliferation in Karpas422 compared to TMD8, with a roughly 5-10 fold lower GI_50_ (Figure 1B). By contrast, both mTOR inhibitors showed slightly greater activity in TMD8 (SF 1A). To confirm that AKT inhibition is not ineffective due to a lack of AKT signaling in resistant lines, we assessed changes in phosphorylation of two AKT substrates, PRAS40 and GSK3β, in response to GSK690693 in four DLBCL lines. All lines showed a similar dephosphorylation of both substrates, demonstrating that AKT signaling is intact in all four cell lines (Figure 1C). We also assessed AKT activation loop phosphorylation at T308, which is essential for AKT activity. While, ABC lines showed lower basal AKT phosphorylation, AKT was hyperphosphorylated in response to AKTi in all lines, demonstrating that this pathway is active. Additionally, we assessed expression of all AKT isoforms (AKT1/2/3) and PTEN across the panel. Clustering analysis showed that AKT1 expression did not discriminate between ABC and GCB lines (SF 2). Surprisingly, higher expression of AKT2 and AKT3 was associated with the ABC subtype. This may account for the fact that resistance to MK2206 is particularly apparent in TMD8 cells. MK2206, unlike catalytic inhibitors of AKT, inhibits AKT3 to a lesser extent than AKT1 or AKT2 [18]. PTEN expression was not correlated with AKTi sensitivity (*p* = 0.886; SF2).

### Distinct mechanisms of mTOR regulation determines sensitivity to AKT inhibitors

**Figure 2:**
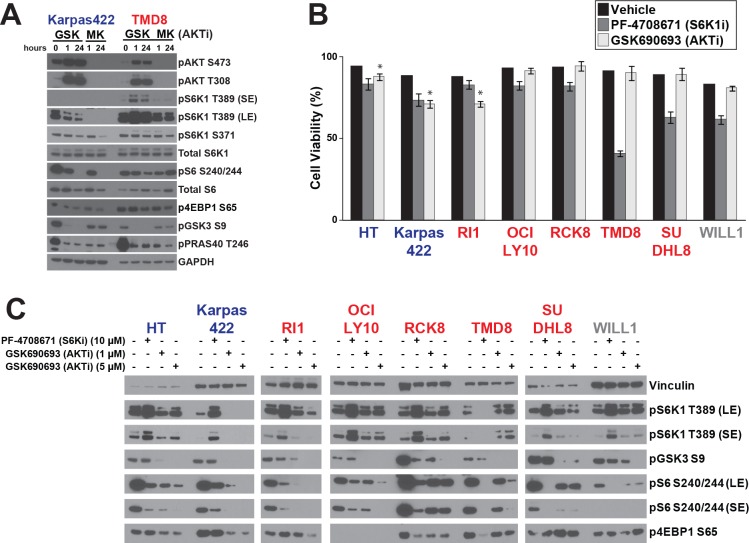
Distinct regulation of S6K1 signaling in DLBCL subtypes **A.** DLBCL lines were treated with GSK690693 (5μM) and MK2206 (5μM) for 1h or 24h before Western blotting. **B.** Cell lines were treated with PF-4708671 (10μM) or GSK690693 (5μM) and cell viability was measured after 72h by trypan blue staining followed by Cellometer reading (*n* = 3). Asterisk indicates *p* < 0.05 **C.** Cell lines were treated with the indicated compounds for 24h. ABC cells are colored in red. GCB are colored in blue. Intermediate cells are colored in gray.

Our observation that all DLBCL lines tested were similarly sensitive to mTOR inhibitors while showing widely divergent sensitivities to AKTi raised the question of whether AKT is the primary regulator of mTOR signaling in DLBCL. To gain greater mechanistic insight into the effects of AKTi on downstream signaling, we decided to compare AKTi sensitive and resistant lines for qualitative differences in downstream signaling pathways. For this comparison, we defined a GI_50_ value of 1μM as the cutoff point. We treated Karpas422 (sensitive) and TMD8 (resistant) with GSK690693 and MK2206 and assessed the phosphorylation of various direct and indirect targets of AKT signaling. As expected, both cell lines showed hyperphosphorylation of AKT in response to the catalytic inhibitor GSK690693 [19] and loss of AKT phosphorylation in response to the allosteric inhibitor MK2206 (Figure 2A). Both cell lines also showed inhibition of AKT substrate phosphorylation (pGSK3 and pPRAS40). However, we noted a striking discrepancy in the response of mTOR substrates to AKTi. In Karpas422, AKTi inhibited phosphorylation of the direct mTOR substrates 4EBP1 and S6K1, as well as the indirect substrate S6. This is consistent with the established view of AKT as the primary regulator of mTOR signaling in most contexts. However, AKTi treatment of TMD8 resulted in little to no dephosphorylation of these substrates. In fact, GSK690693 treatment actually showed a dramatic increase in S6K1 phosphorylation in TMD8 cells. These data suggest that mTOR signaling may not be primarily regulated by AKT in TMD8.

We expanded our analysis by comparing the effects of an S6K1-specific inhibitor, PF-4708671 [20] with GSK690693 across eight DLBCL lines of varying AKTi sensitivity. We observed that AKTi-sensitive cell lines showed a small but statistically significant (*p* < 0.05) drop in viability with GSK690693 that was not seen in AKTi-resistant lines (Figure 2B). PF-4708671, by contrast showed a reduction in viability in all cell lines that was greater in the majority of AKTi-resistant lines. We interrogated AKT/mTOR signaling by Western blot and found that AKT-sensitive lines (HT, Karpas422 and Ri1) showed inhibition of S6K1 phosphorylation by GSK690693, while AKTi-resistant lines (OCI-LY10, RC-K8, TMD8, SUDHL8 and WILL-1) showed no downregulation of pS6K1 or, in some cases, increased phosphorylation (Figure 2C).

When we examined S6 phosphorylation, we found a systematic difference between AKTi-sensitive and resistant lines (Figure 2C). In sensitive lines, the AKT inhibitor caused greater dephosphorylation relative to PF-4708671, whereas in AKT-resistant lines PF-4708671 was more effective in dephosphorylation relative to GSK690693. Therefore, while the extent of dephosphorylation seen with these inhibitors varies significantly by cell line, the relative potency of S6 dephosphorylation between AKT and S6K1 inhibitors can distinguish AKTi-sensitive and resistant lines. These data suggest a difference in S6K1/S6 regulation between these two clusters of DLBCL lines.

### Regulation of mTOR by BCR signaling

**Figure 3:**
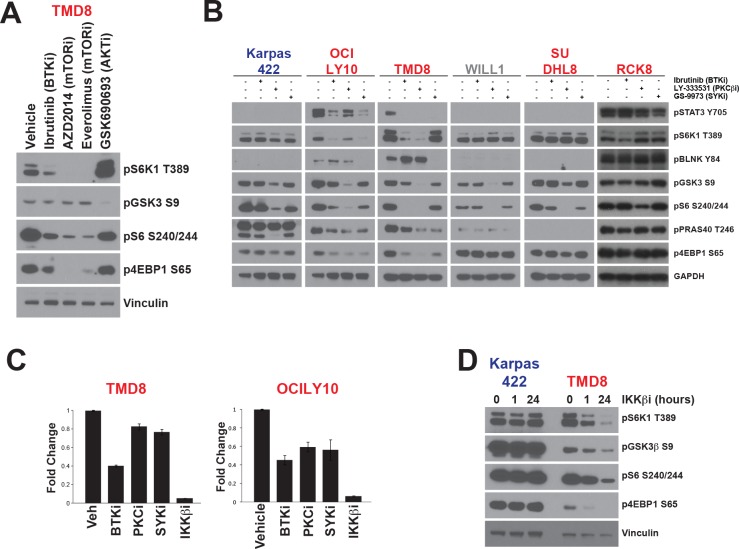
mTOR signalling is activated independently of AKT in ABC-DLBCL **A.** TMD8 cells were treated for 24h hours with ibrutinib (10nM), AZD2014 (200nM), everolimus (100nM) or GSK690693 (5μM). **B.** Cell lines were treated for 24 hours with inhibitors of BTK (ibrutinib, 10nM), PKCβ (LY-333531, 4μM), SYK (GS-9973, 1μM) **C.** TMD8 and OCILY10 cells stably expressing pGreenFire NF-κB-Luc were treated for 18h with inhibitors at previously indicated concentrations and IKKβ (Bayer inhibitor C_21_H_25_ClN_4_O_2_, 3μM). Luciferase activity was then assayed using the Dual-Glo (*n* = 6). Luminescence was normalized to cell number from viable cell counting by Cellometer. **D.** Cells were treated for 1h or 24h with Bayer IKKβ inhibitor (3μM) before Western blotting. ABC cells are colored in red. GCB are colored in blue. Intermediate cells are colored in gray.

Phosphorylation of S6K1 on T389 in its activation loop is essential to its activity [21]. mTOR is a well-established regulator of S6K1 through phosphorylation of this site. We confirmed that mTOR activity is essential for S6K1/S6 signaling in AKTi-sensitive and resistant lines (SF1B, Figure 3A, and [9]). However, given that T389 phosphorylation is only regulated by AKT in sensitive lines, there must be an alternative mechanism regulating S6K1 in those DLBCL lines that were resistant to AKTi. Since most AKTi-resistant lines are ABC-DLBCL, we hypothesized that mTOR signaling in these cell lines is regulated through BCR signaling to bypass the requirement for AKT. We previously published evidence that ibrutinib, a highly specific inhibitor of the tyrosine kinase and BCR signaling component BTK, could downregulate mTOR signaling in ABC-DLBCL [9]. We compared the ability of ibrutinib, inhibitors of mTOR, and GSK690693 to block mTOR substrate phosphorylation in ABC-subtype TMD8 cells (Figure 3A). Ibrutinib could significantly reduce S6K1 pT389 as well as S6 and 4EBP1 phosphorylation, although to a lesser extent than mTOR inhibitors. By contrast, GSK690693 treatment increased pT389 and had little or no effect on pS6 or p4EBP1. Unlike AKTi, the mTOR inhibitors did not affect pGSK3β, demonstrating no evidence of AKT inhibition, and ibrutinib showed a variable and lesser effect on pGSK3β (Figure 3A, 3B). These data confirm our previous results and, further, suggest that BTK might regulate mTOR independently of AKT. Since BTK and AKT inhibitors have opposite effects on pS6K1, BTK may replace AKT as an upstream regulator of mTOR signaling in ABC-DLBCL.

Given the effect of ibrutinib on mTOR signaling, we extended our analysis to determine whether other kinases involved in BCR signaling could also regulate mTOR. We compared ibrutinib with specific inhibitors of SYK (GS-9973) [22], an upstream regulator of BTK, and PKCβ (LY-333531)[23] which lies downstream of BTK in the regulation of NF-κB. At the concentrations used, ibrutinib and GS-9973 are reported to be highly specific [22, 24]. Less information exists for LY-333531, although it has been shown to be highly selective for PKCβ over other PKC isoforms [23]. We used pBLNK as a proximal marker for BCR signaling and pSTAT3 a marker for signaling downstream of the BCR. As expected, only TMD8 and OCILY10 showed strong phosphorylation of these markers, consistent with the presence of activating mutations in CD79 and all inhibitors blocked pSTAT3, while only SYK inhibition downregulated pBLNK (Figure 3B).

In all lines tested, other than RCK8, LY-333531 strongly inhibited phosphorylation of GSK3β and S6, and inhibited PRAS40 and 4EBP1 phosphorylation in some cell lines (Figure 3B). This suggests that PKCβ may be an essential regulator of AKT and mTOR signaling in DLBCL, although we cannot entirely rule out an off-target effect on AKT, as both AKT and PKCβ are both AGC family kinases. However, LY-333531 had similar effects in ABC and GCB lines and so cannot explain our observation of AKT-independent activation of mTOR signaling in ABC lines.

GS-9973 downregulated PRAS40 and 4EBP1 phosphorylation in TMD8 and OCI-LY10, but did not potently inhibit phosphorylation of other AKT/mTOR substrates. Ibrutinib strongly reduced phosphorylation of 4EBP1, S6, and S6K1 in OCI-LY10 and TMD8 but not in any other cell lines. Even the other ABC lines tested (RI1, SUDHL8, and RCK8) did not show a significant reaction to BTK or SYK inhibition at the concentrations tested. TMD8 and OCILY10 have activating mutations in one of the CD79 subunits of the BCR that are not present in the other cell lines tested [25] as well as a mutation in MyD88 (L265P) which has been demonstrated to directly activate BTK [26]. Therefore, BTK and SYK may be required for mTOR signaling in cell lines with active BCR signaling.

Our observation that inhibitors of BCR signaling could block mTOR in ABC lines raised the question of how BCR and mTOR signaling are integrated in this context. The primary output of BCR signaling that has been linked to DLBCL pathogenesis is NF-κB. A key regulator of NF-κB, the kinase IKKβ, has previously been shown to regulate mTOR through phosphorylation of TSC1 [27]. To explore whether NF-κB could be the link between BCR and mTOR, we used a luciferase reporter system to measure NF-κB activity in OCI-LY10 and TMD8 [9]. In both lines, ibrutinib inhibited luciferase activity to a similar extent and inhibitors of SYK and PKCβ were more effective in blocking NF-κB in OCILY10 than TMD8 (Figure 3C). Since the PKCβ inhibitor could affect mTOR signaling even in GCB lines, which do not show activation of NF-κB, we discounted this mechanism as an explanation for the effects of this inhibitor. However, the extent of NF-κB inhibition by the SYK inhibitor GS-9973 correlated with downregulation of mTOR signaling in these cell lines. This result supported a mechanism whereby IKKβ links BCR signaling to mTOR. To test this hypothesis, we treated TMD8 and Karpas422 cells with a specific IKKβ inhibitor [28]. This compound had no effect on mTOR signaling in Karpas422, consistent with the lack of NF-κB activation in this GCB line (Figure 3D). In stark contrast to the results in Karpas422, IKKβ inhibition strongly downregulated phosphorylation of both AKT and mTOR substrates in TMD8. We also confirmed that changes in phosphoprotein abundance as a result of treatment with these inhibitors is not the result of changes in total protein abundance (SF4). These data support a model through which ABC lines with activating mutations in BCR require NF-κB signaling to activate mTOR.

### PIM2 can promote resistance to AKTi

**Figure 4:**
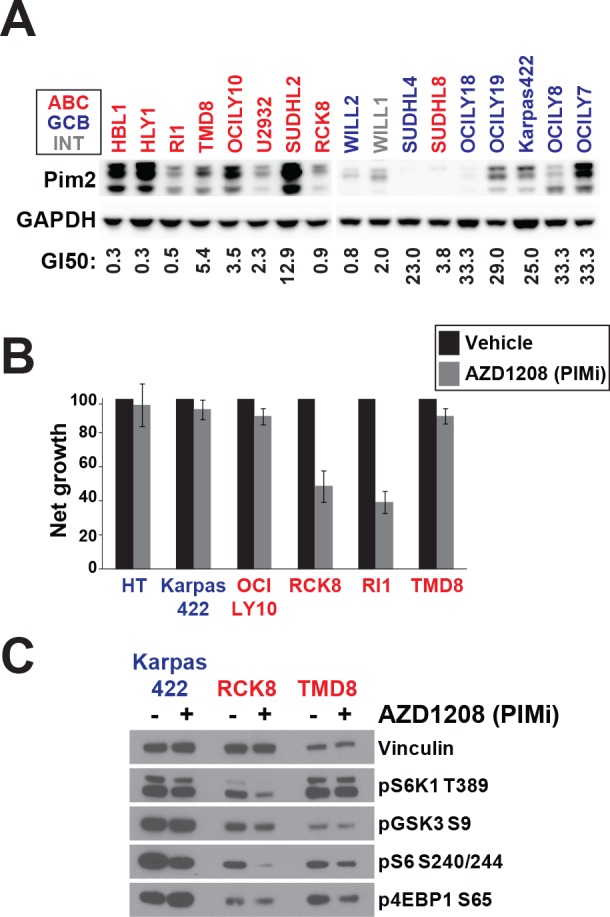
PIM can regulate AKT-independent mTOR signalling **A.** PIM2 protein expression was assayed by Western blot. GI_50_ values for AZD1208 were derived from 72h day Alamar Blue assay. **B.** Net growth was measured over 72h by viable cell counting using the Cellometer system (*n* = 3) **C.** Cell lines were treated for 24h with AZD1208 (1μM) before Western blotting. ABC cells are colored in red. GCB are colored in blue. Intermediate cells are colored in gray.

PIM2 has been reported to be upregulated in some ABC-DLBCL lines and may be necessary for their proliferation and survival [29]. PIM kinases share overlapping substrate specificities with AKT kinases and may regulate mTOR independently of AKT. We investigated whether PIM2 upregulation is associated with resistance to AKTi. We profiled sensitivity to AZD1208, a selective pan-PIM inhibitor [30], across the DLBCL panel (Figure 4A). In agreement with previous results, we found that ABC-DLBCL had generally lower GI_50_ values than GCB-DLBCL and that sensitivity to AZD1208 was correlated with ABC identity (*p* = 0.014)(Supplemental Table 1). We also measured PIM2 protein expression across the DLBCL panel and confirmed that ABC-DLBCL show higher expression on average, although there is significant variability within both subtypes (Figure 4A). Across the panel, AZD1208 sensitivity was correlated with mRNA expression of PIM2 (*p* = 0.034)(Supplemental Table 2). However, it was clear that some ABC lines express high levels of PIM2 without being sensitive to the compound and the correlation between PIM2 protein expression and sensitivity was very weak (least squares regression = −0.098). This indicates that factors other than PIM2 expression affect AZD1208 sensitivity.

We selected several DLBCL lines and confirmed their sensitivity to AZD1208 by measuring net growth after single agent treatment (Figure 4B). Among ABC-DLBCL, RCK8 and RI1 were particularly sensitive to AZD1208 treatment, in agreement with our profiling data. TMD8, which expressed higher levels of PIM2 than RCK8 and Ri1, was resistant to AZD1208, again suggesting that PIM2 expression does not solely determine sensitivity. We hypothesized that the ability of AZD1208 to block proliferation would correlate with its effect on mTOR signaling. We assessed phosphorylation of mTOR targets in three DLBCL lines treated with AZD1208 (Figure 4C). We found that AZD1208 did not affect the AKT substrate GSK3β and showed a slight inhibition of 4EBP1 phosphorylation in both ABC lines. Interestingly, a differential response to PIM inhibition was observed. S6K1 and S6 phosphorylation were downregulated in the sensitive line RCK8 but not in TMD8 or Karpas422. These data suggest that inhibition of S6K1/S6 is a critical pharmacodynamics surrogate for growth inhibition by agents specificially targeting mTOR signaling.

### Combined inhibition of multiple kinases efficiently blocks mTOR signaling

Our data have suggested that multiple kinases can regulate mTOR signaling to influence proliferation and survival in DLBCL. Agents inhibiting AKT, PIM, BTK, or S6K1 are each effective in a subset of DLBCL. We pursued a strategy of combining these inhibitors to achieve greater efficacy. Karpas422 cells showed particular sensitivity to AKTi (SF3A) but only underwent a low level of cell death in response to single agent treatment. Combined inhibition of AKT and S6K in this line caused greater cell death relative to either inhibitor alone (SF3B). When S6 phosphorylation was assessed, we found that combined inhibition resulted greater dephosphorylation than the single agents (SF3C). We made use of the dual AKT/S6K inhibitor AT7867 [31] to see if this was a general phenomenon in DLBCL lines. AT7867, unlike S6K or AKT inhibitors, resulted in complete growth inhibition of five ABC and GCB lines and induced cell death at higher concentrations (SF3D). These data support a model in which AKT and S6K are partially redundant in DLBCL and combined inhibition can overcome intrinsic resistance to AKTi in ABC-DLBCL.

**Figure 5:**
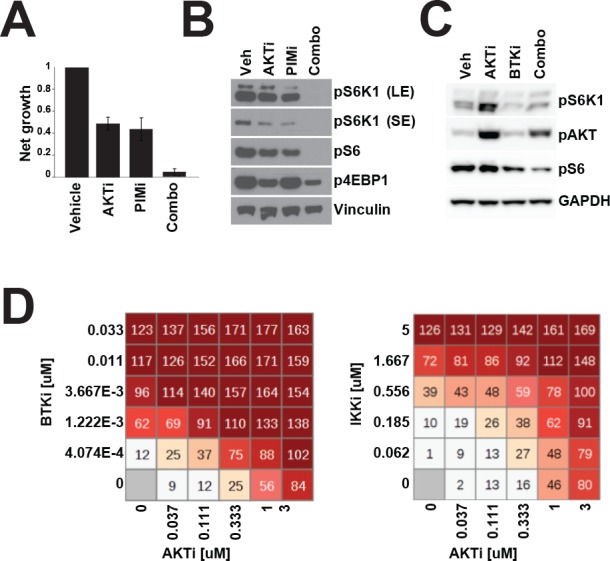
Combination therapy more effectively inhibits mTOR signalling **A.** RI1 cells were treated with GSK690693 (1μM) and AZD1208 (1μM) and proliferation was measured over 72h by CellTiterGlo. **B.** As in **A.** but cell were treated for 24h before Western blotting. **C.** TMD8 cells were treated with GSK690693 (1μM) and ibrutinib (10nM) for 24 hours before Western blotting. **D.** 72 hour viability was measured in TMD8 by Alamar Blue assay after treatment with ibrutinib and GSK690693 at the indicated concentrations.

Based on our data, PIM or AKT inhibition can downregulate mTOR in some DLBCL cell lines. Using our GI_50_ values for AZD5363 and AZD1208, we chose to test this combination in Ri1 cells, which are moderately sensitive to both AKT and PIM inhibitors as single agents. Simultaneous treatment with both AKT and PIM inhibitors resulted in nearly complete growth inhibition in Ri1 cells (Figure 5A), a significant combination benefit over the single agents. Western blot analysis of Ri1 showed that either AKT or PIM inhibition alone resulted in moderate inhibition of pS6K1 and pS6 whereas the combination treatment completely blocked phosphorylation of these proteins, but not 4EBP1 (Figure 5B). We also tested this combination in HBL1 cells, which are highly resistant to AKTi. Synergy was observed although to a much lesser degree than in RI1 (SF5).

Finally, we tested whether inhibition of BTK could overcome the AKTi-resistant phenotype exhibited by ABC-DLBCL lines. We tested the combination of ibrutinib and GSK690693 in TMD8 cells and found that ibrutinib could not only downregulate S6K1 phosphorylation, but also completely block AKTi-induced hyperphosphorylation of S6K1 (Figure 5C). The combination was also more effective at dephosphorylating S6. We tested whether there was a synergistic interaction between AKTi and ibrutinib in this cell line and found clear evidence of synergy (Figure 5D; synergy score: 14.7). When we measured the synergy between AKTi and IKKi in TMD8, we found only very low synergy (Figures 5D and SF5; synergy score: 3.6). We confirmed that the combination of IKK and AKT inhibition is only weakly synergistic in OCILY10 (SF5; synergy score: 3.9). These data (along with the data shown in Figure 3) suggest that in ABC-DLBCL, NF-κB and mTOR signaling converge at IKK and that BTK serves to link BCR signaling to mTOR, perhaps through IKK.

## DISCUSSION

Significant evidence supports the examination of PI3K/AKT/mTOR signalling as a therapeutic target in DLBCL. However, little information exists on the most effective strategy for targeting this pathway in DLBCL or on the differential response of DLBCL subtypes. Here we demonstrate that mTOR inhibitors as a class are broadly effective in arresting proliferation of DLBCL lines, regardless of ABC or GCB classification. However, DLBCL lines differ greatly in their sensitivity to AKT inhibitors, both ATP-competitive and allosteric. Furthermore, we show a correlation between the ABC subtype and resistance to inhibition of AKT. The finding that there is a clear uncoupling between sensitivity to mTOR and AKT inhibitors is surprising given that AKT has been well established has a regulator of mTORC1 in response to extracellular stimuli [32]. Therefore, we explored the regulation of mTOR in DLBCL. We found that GCB lines, some of which show activation of AKT through mutation or deletion of PTEN, preserve canonical signalling between AKT and mTOR and that AKT inhibitors can downregulate mTORC1 substrate phosphorylation in these cells. ABC lines, however, show a regulation of mTOR that is quite distinct from this described mechanism. In these lines, AKT inhibitors have a lesser effect on the phosphorylation of translational inhibitors S6 and 4EBP1. Even more strikingly, phosphorylation of S6K1, an mTOR substrate and the immediate upstream regulator of S6, is downregulated by AKT inhibitors in GCB-DLBCL but is either entirely unaffected or upregulated in ABC-DLBCL. A clear correlation among DLBCL lines is seen between dephosphorylation of S6 by AKT inhibitors and sensitivity. And surprisingly, ABC-DLBCL lines that show a lesser dependence on AKT for S6 phosphorylation show a greater dependence on S6K1.

These data suggested alternative modes of mTOR, and particularly S6K1, activation in ABC-DLBCL. We find that in some ABC-DLBCL lines, the tyrosine kinase BTK is required for mTOR activation and may act through NF-κB signaling. In other lines, PIM2 is required for S6K1/S6 signalling, in agreement with data showing that PIM kinases share substrates with AKT kinases [33]. These mechanisms can promote AKT-independent but mTOR-dependent activation of translational machinery [34]. We have also established that there is a strong correlation between ABC identity and AKTi resistance in ABC-DLBCL. Given these data, it is plausible that the primary explanation for AKTi resistance in ABC lines is the prevalence of BCR pathway mutations (including CD79 and MyD88 mutations) that render mTOR activation independent of AKT. In ABC lines without these mutations, PIM2 upregulation may serve the same purpose. Our data also suggests that multiple pathways can redundantly regulate mTOR activation in DLBCL lines. For example, AKT and PIM2 can both regulate mTOR in RI1 and combined inhibition is required to completely downregulate S6K signaling in this line.

In summary, our data point toward the existence of multiple mechanisms for the regulation of mTOR in DLBCL (SF6). The sensitivity of DLBCL lines to AKT inhibitors appears to be determined by the genetic background of the cell line, which may help to determine which of these mechanisms is dominant (Supplemental Table 4). Therefore, understanding the classification of DLBCL may be very important for effective targeting of mTOR signalling and therapeutic use of PI3K/AKT/mTOR pathway inhibitors could be influence by mutational analysis of patient tumors. Given the increasing importance of PI3K inhibition in targeted therapies for lymphoma, this may be of clinical interest [35]. We also show that combination therapy to inhibit more than one kinase involved in mTOR signaling can more effective than targeting a single kinase.

## MATERIALS AND METHODS

### Western blots and drug treatments

Western blots were performed as previously described [9]. Antibodies for AKT, GSK3, S6, 4EBP1, S6K1, GAPDH, vinculin, and STAT3 were purchased from Cell Signaling Technology. PRAS40 antibody was purchased from Invitrogen. BLNK antibody was purchased from BD Biosciences. GSK690693, MK2206, everolimus, rapamycin, PF-4708671, AT7867, GS-9973, and ibrutinib were purchased from Selleck. LY-333531 was purchased from Tocris. AZD2014, AZD5363, AZD1208, and C_21_H_25_ClN_4_O_2_ were synthesized by AstraZeneca.

### Proliferation and viability assays

Alamar blue assays were performed as previously described [9]. Net growth proceeded as follows: cells were seeded at predetermined densities, dosed the next day (D0), and read on Day 3. Net growth was calculated using the following formula: (Experimental reading - D0 reading)/(DMSO reading - D0 reading). CellTiterGlo experiments were performed as Alamar blue assays, except CellTiterGlo reagents was added 1:1 to medium containing cells and plates were incubated for 10 minutes at room temperature before luminescence was read on a Tecan system. Cell viability was measured by trypan blue staining (1:2 dilution of cells in 0.1% trypan blue) followed by automated cell counting using the Cellometer system. 6×6 matrix-based synergy assays were performed as previously described [9].

### Luciferase assays

Cells were plated at 500,00/ml and treated for 18 hours with indicated compounds. Cells were then collected by centrifugation and normalized by Cellometer reading. Cells were plated at 100,000/ml in 96-well white bottom plates (100μl). Dual-Glo luciferase substrate (Promega) was added at a 1:1 ratio and plates were incubated at room temperature for 10 minutes before reading on a Tecan system.

### Bioinformatics

Cell of origin (COO) values were derived as previously described [15]. Statistical tests were performed in R version 3.1.1. The expressions of AKT isoforms were measured using microarray data. Representative probesets were selected to represent each isoform (207163_s_at for AKT1, 225471_s_at for AKT2 and 212607_at for AKT3). Measured signals were log2 transformed before the analysis. The transformed values were then clustered using complete linkage method with Euclidean distance measure. The heatmap was prepared using R (heatmap.2 method in package gplots). The Affymetrix U133 plus 2 cell line expression data can be found at GEO accession GSE57083.

GI_50_ values were transformed into pGI_50_ using -log10(GI_50_), where value of 6.0 corresponds to 1.0 μM. Higher values indicate higher sensitivities. The transformed values were then clustered using complete linkage method with Euclidean distance measure. The heatmap was prepared using R (heatmap.2 method in package gplots).

## SUPPLEMENTARY MATERIAL TABLES AND FIGURES










