# Supplementary material for: Differential regulation of mTOR signaling determines sensitivity to AKT inhibition in diffuse large B cell lymphoma
Source: Oncotarget. 2016 Jan 27;7(8):9163–74. doi: 10.18632/oncotarget.7036 (PMC4891033; doi:10.18632/oncotarget.7036)
Supplement: Supplementary file 1 [file oncotarget-07-9163-s001.pdf]

Differential regulation of mTOR signaling determines sensitivity to AKT inhibition in diffuse large B cell lymphoma

Supplementary Material

Supplemental Table 1: Analysis of drug sensitivity across a DLBCL panel. DLBCL lines were assigned to the GCB or ABC subtype or intermediate using cell of origin (COO) values. GI_{50} values for inhibitors of AKT, PIM, and mTOR were correlated with COO values using R.

Supplemental Table 2: Association between PIM expression and AZD1208 sensitivity. Microarray expression data for PIM1, PIM2, and PIM3 was correlated with sensitivity to AZD1208 and AZD5363.

Supplemental Table 3: List of inhibitors used.

Supplemental Table 4: Relevant mutations identified in DLBCL lines. Known genetic alterations in genes relating to BCR signaling, NF- κB, and PI3K signaling, as well as highly recurrent mutations in MYC and BCL2 are shown for each cell line.

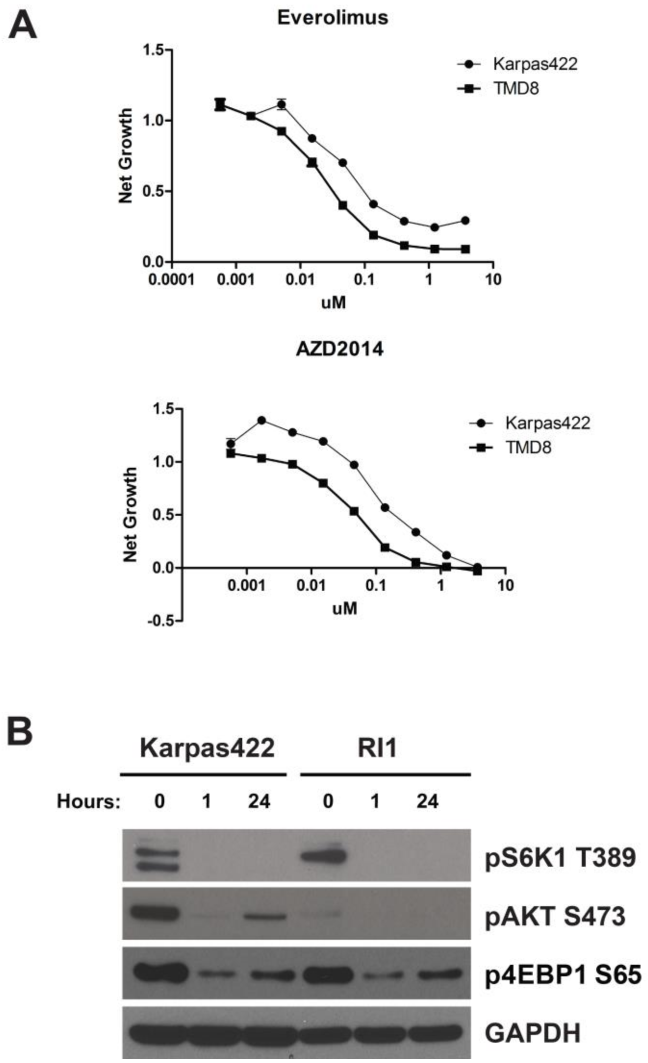

Supplemental Figure 1: mTOR signalling in DLBCL. (A) Dose response curves were generated using a 72h CellTiterGlo assay. (B) Cells lines were treated for the indicated times with AZD2014 (200nM).

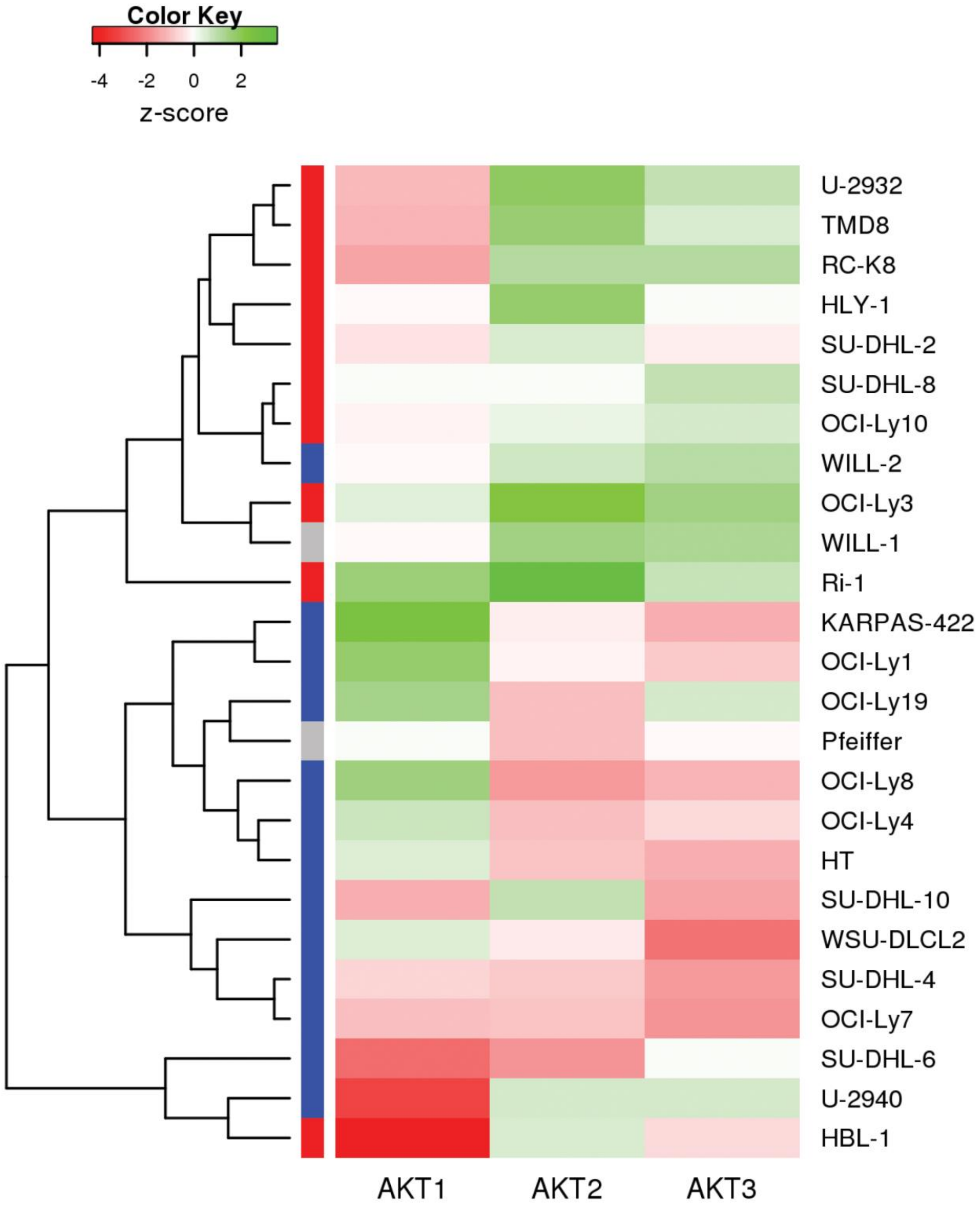

Supplemental Figure 2: Expression of AKT isoforms in DLBCL. Microarray expression data was used to cluster DLBCL lines according to the expression of AKT1, AKT2, and AKT3.

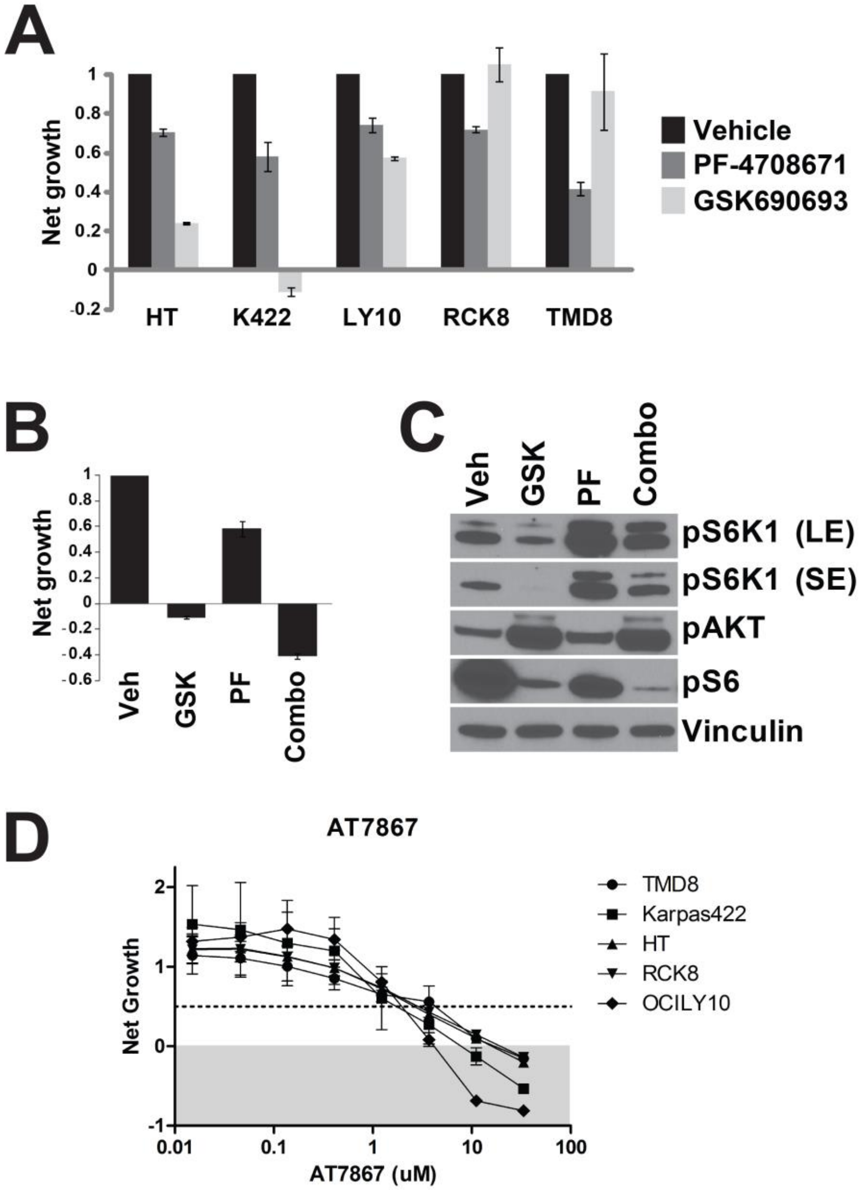

Supplemental Figure 3: Comparison of AKT and S6K1 inhibitors. (A) Net growth calculated using 72h CellTiterGlo assay. GSK690693 = 1μM; PF-4708671 = 10μM. (B) Karpas422 cells were treated with GSK690693 (1μM) and PF-4708671 (10μM) and proliferation was measured over 72h by CellTiterGlo. (C) As in (B) but cell were treated for 24h. (D) Net growth calculated using 72h CellTiterGlo assay.

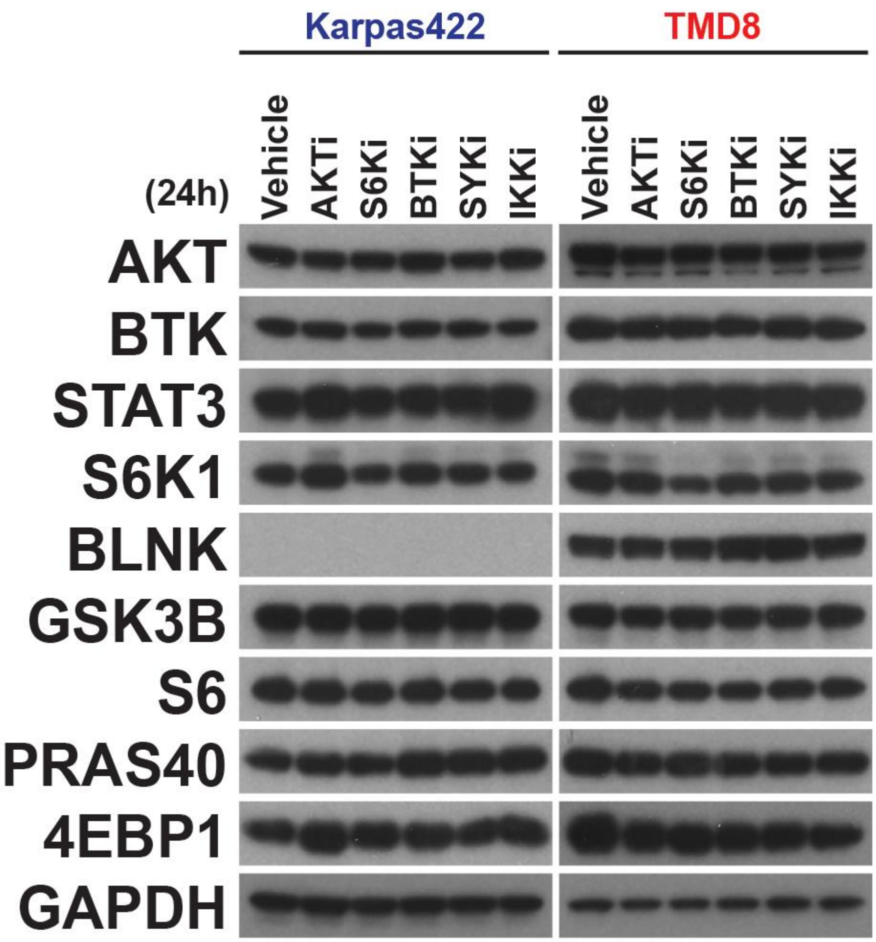

Supplemental Figure 4: Total protein abundance does not change. Karpas422 and TMD8 cells were treated for 24h using the following inhibitors: GSK690693 (1µM), PF-4708671 (10 µM), ibrutinib (10nM), GS-9973 (1µM), IKK inhibitor (3µM).

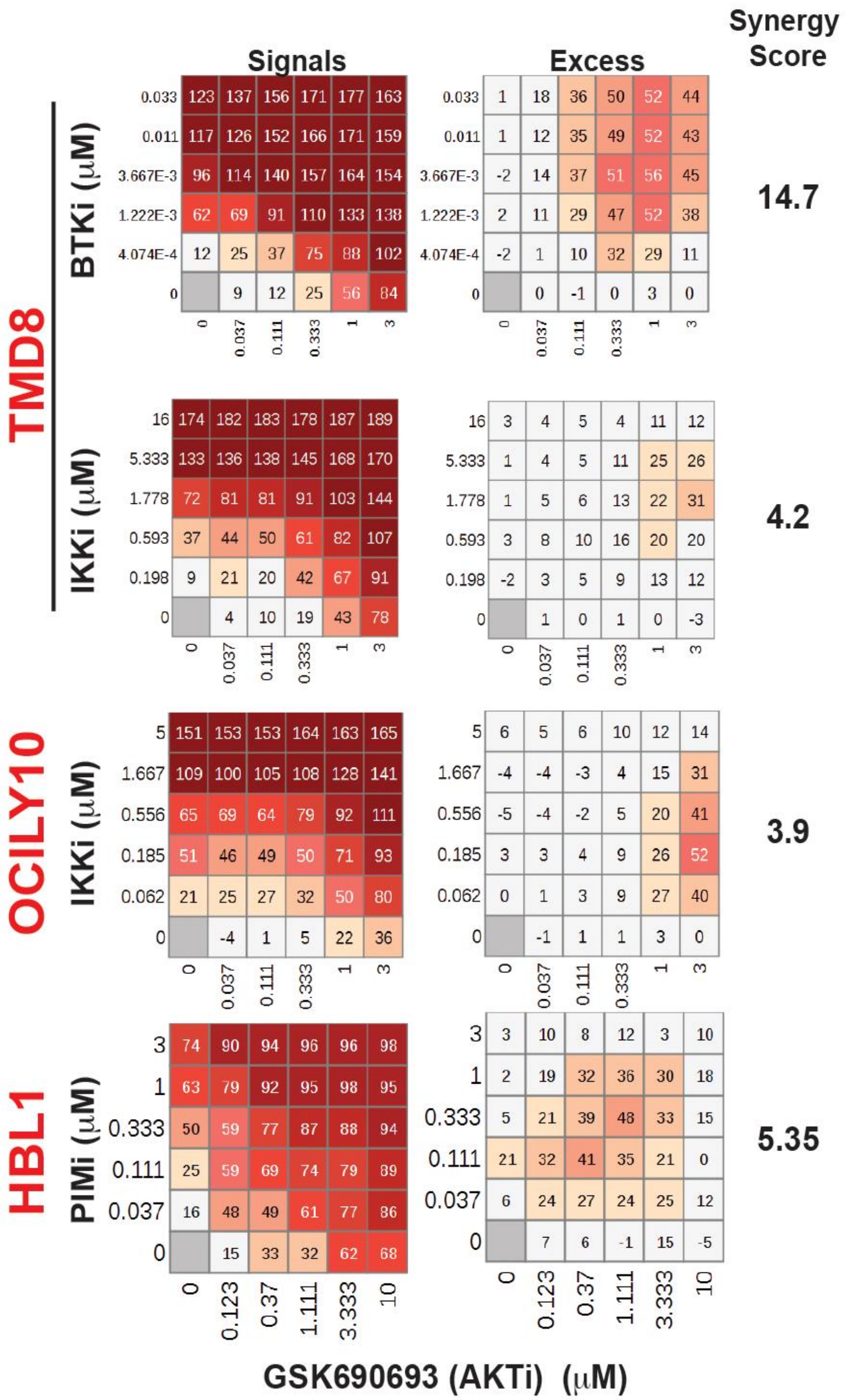

Supplemental Figure 5: Drug combinations in ABC-DLBCL. Synergy experiments were performed with the indicated compounds using a 72h Alamar Blue assay and synergy scores were calculated as described in the Materials and Methods section.

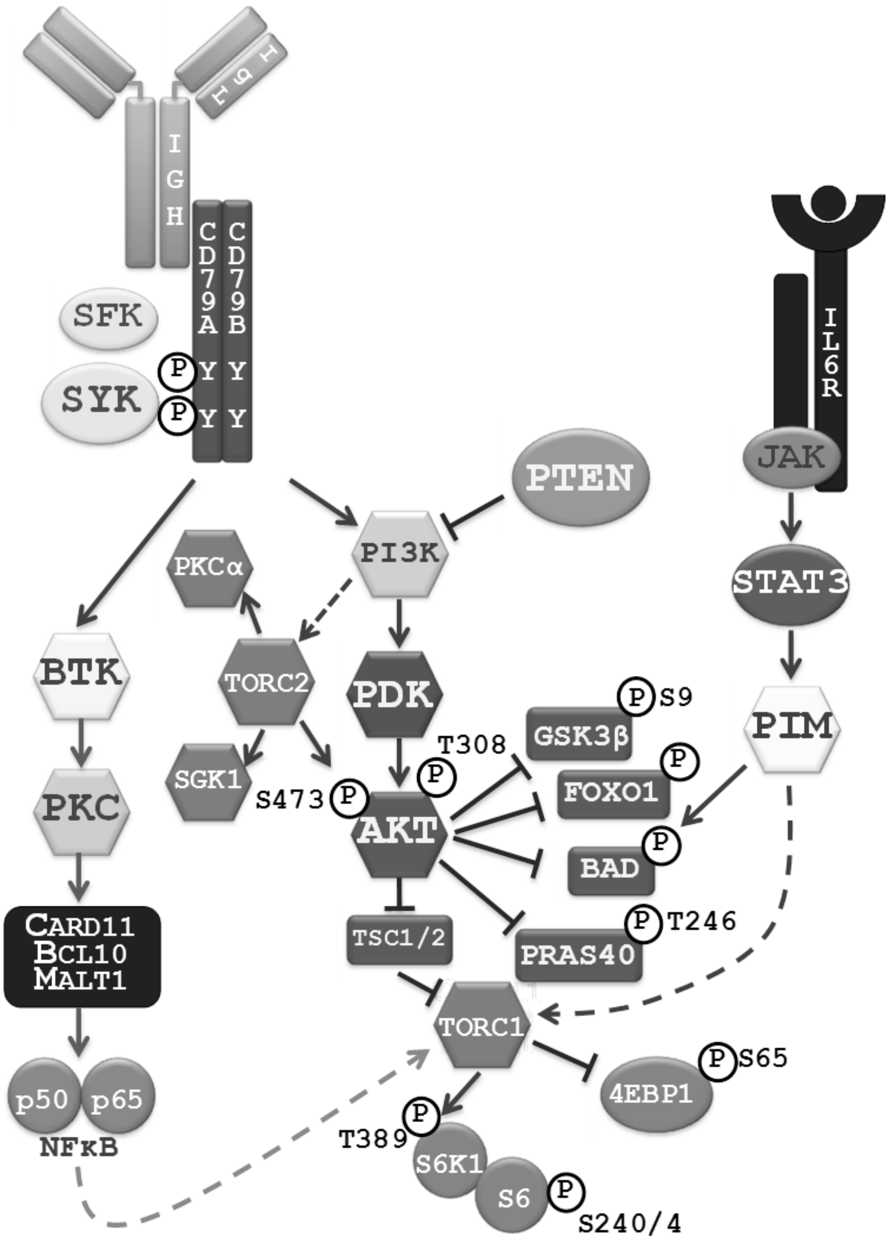

Supplemental Figure 6: Proposed model for regulation of mTOR signaling in DLBCL. These data support a model whereby mTOR activation is regulated by multiple inputs in DLBCL, including AKT, BCR/NF- κB signaling, and PIM. The balance of these inputs may be determined by the genetic background of the cell line or tumor, with ABC-type lines carrying activation mutations in CD79A/B requiring BTK signaling, wheras other ABC-type lines expressing high levels of PIM2 do not require this pathway. GCB-DLBCL appear to rely on the canonical signaling pathway through AKT to mTOR.
